# Supplementary material for: Venous Thromboembolism in Cancer Patients Undergoing Chemotherapy: A Systematic Review and Meta-Analysis
Source: Diagnostics (Basel). 2022 Nov 25;12(12):2954. doi: 10.3390/diagnostics12122954 (PMC9777086; doi:10.3390/diagnostics12122954)

Supplementary Information (SI)

Venous thromboembolism in Cancer Patients Undergoing Chemotherapy: A Systematic Review and Meta-Analysis

Authors: Ming-Yee Sun and Sonu M. M. Bhaskar*

***Correspondence to**: Dr Sonu M. M. Bhaskar, MD PhD FANA
Email: **Sonu.Bhaskar@globalhealthneurolab.org**
Ph: +61 (02) 873 89179 Fax: +61 (02) 8738 3648
Department of Neurology & Neurophysiology, Neurophysiology Level 1, Clinical Sciences Building, Elizabeth St, Liverpool Hospital, NSW 2170

List of Contents

1. **Search Strategy** (Keywords/MeSH Terms)
2. **PRISMA Checklist**
3. **Supplemental Tables**
   a. **Supplemental SI Table S1:** Modified Jadad Analysis for Methodological Quality
   b. **Supplemental SI Table S2:** Funding bias scores for studies
4. **Supplemental Figures:**
   a. **Supplemental SI Figure S1:** Forest plot showing the estimated pooled prevalence of VTE in bladder cancer patients
   b. **Supplemental SI Figure S2:** Forest plot showing the estimated pooled prevalence of VTE in brain cancer patients
   c. **Supplemental SI Figure S3:** Forest plot showing the estimated pooled prevalence of VTE in breast cancer patients
   d. **Supplemental SI Figure S4:** Forest plot showing the estimated pooled prevalence of VTE in colorectal cancer patients
   e. **Supplemental SI Figure S5:** Forest plot showing the estimated pooled prevalence of VTE in gastric cancer patients
   f. **Supplemental SI Figure S6:** Forest plot showing the estimated pooled prevalence of VTE in lung cancer patients

g. **Supplemental SI Figure S7:** Forest plot showing the estimated pooled prevalence of VTE in lymph cancer patients

h. **Supplemental SI Figure S8:** Forest plot showing the estimated pooled prevalence of VTE in mesothelial cancer patients

i. **Supplemental SI Figure S9:** Forest plot showing the estimated pooled prevalence of VTE in ovarian cancer patients

1. Search Strategy (Keywords/MeSH Terms)

Search Strategy used for PubMed

(("Cancer" OR "Carcinoma" OR "Breast Cancer" OR "lymphoma" OR "Hodgkin's Lymphoma" OR "Non-Hodgkin's lymphoma" OR "Germ cell tumor" OR "Bladder cancer" OR "Lung cancer" OR "Colorectal cancer" OR "Pancreatic cancer" OR "Bone Cancer" OR "Stomach Cancer" OR "Glioblastoma" OR "Brain Cancer" OR "Malignant" OR "meningiomas" OR "schwannomas" OR "pineal gland tumors" OR "pituitary tumors" OR "astrocytomas" OR "Tumor" OR "tumour" OR "Benign Tumor" OR "malignant tumor" OR "Neoplasm" OR "Metastasis" OR "Hamartoma" OR "Carcinoma" OR "Sarcoma" OR "Blastoma" OR "Papilloma" OR "Adenoma" OR "Adenocarcinoma" OR "Paraneoplastic syndrome") OR ("Chemotherapy" OR "Chemotherapeutic" OR "Cisplatin" OR "neoplatin" OR "cismaplat" OR "cis-diamminedichloroplatinum" OR "CDDP" OR "Carboplatin" OR "Paraplatin" OR "Bevacizumab" OR "L-asparaginase" OR "Cyclophosphamide" OR "5-fluorouracil" OR "vinorelbine" OR "CMF" OR "Gemcitabine" OR "FOLFOX" OR "Doxorubicin" OR "Methotrexate" OR "Ifosfamide" OR "Etoposide" OR "MAP" OR "MAPIE" OR "Cyclophosphamide" OR "vincristine" OR "Epirubicin" OR "capecitabine" OR "Bleomycin" OR "prednisolone" OR "Mustine" OR "procarbazine" OR "vinblastine" OR "dacarbazine" OR "Docetaxel" OR "dexamethasone" OR "oxaliplatin")) AND ("Clot" OR "Thrombosis" OR "Venous Thromboembolism" OR "Pulmonary Embolism" OR "Embolism" OR "Deep Vein Thrombosis" OR "DVT" OR "Femoral Vein Thrombosis" OR "Paget-Schroetter Syndrome" OR "PSS" OR "Superior Vena Cava Thrombosis" OR "Jugular Vein Thrombosis" OR "Cerebral Venous Sinus Thrombosis" OR "Cavernous Sinus Thrombosis" OR "Retinal Vein Occlusion" OR "May-Thurner Syndrome" OR "Budd-Chiari Syndrome" OR “thromboembolism” OR “thromboembolic” OR “VTE” OR “PE”).

Filters: Full text, Clinical Study, Clinical Trial, Clinical Trial Protocol, Phase I, Phase II, Phase III, Phase IV, Comparative Study, Dataset, Evaluation Study, Meta-Analysis, Multicenter Study, Observational Study, Pragmatic Clinical Trial, Randomised Controlled Trial, Review, Systematic Review, Validation Study, Humans, Adult: 18+ years, from 2012-2022

Results: 2643

2. PRISMA-2020 Checklist

Section and Topic	Item #	Checklist item	Location where item is reported
**TITLE**			
Title	1	Identify the report as a systematic review.	1, 4
**ABSTRACT**			
Abstract	2	See the PRISMA 2020 for Abstracts checklist.	2
**INTRODUCTION**			
Rationale	3	Describe the rationale for the review in the context of existing knowledge.	3
Objectives	4	Provide an explicit statement of the objective(s) or question(s) the review addresses.	3
**METHODS**			
Eligibility criteria	5	Specify the inclusion and exclusion criteria for the review and how studies were grouped for the syntheses.	4
Information sources	6	Specify all databases, registers, websites, organisations, reference lists and other sources searched or consulted to identify studies. Specify the date when each source was last searched or consulted.	4
Search strategy	7	Present the full search strategies for all databases, registers and websites, including any filters and limits used.	4, supplementary information

Selection process	8	Specify the methods used to decide whether a study met the inclusion criteria of the review, including how many reviewers screened each record and each report retrieved, whether they worked independently, and if applicable, details of automation tools used in the process.	4, PRISMA Diagram (Figure 1)
Data collection process	9	Specify the methods used to collect data from reports, including how many reviewers collected data from each report, whether they worked independently, any processes for obtaining or confirming data from study investigators, and if applicable, details of automation tools used in the process.	4-5
Data items	10a	List and define all outcomes for which data were sought. Specify whether all results that were compatible with each outcome domain in each study were sought (e.g. for all measures, time points, analyses), and if not, the methods used to decide which results to collect.	4-6
	10b	List and define all other variables for which data were sought (e.g. participant and intervention characteristics, funding sources). Describe any assumptions made about any missing or unclear information.	4-6, Tables 1-3, Supplementary Information
Study risk of bias assessment	11	Specify the methods used to assess risk of bias in the included studies, including details of the tool(s) used, how many reviewers assessed each study and	4, Supplementary Information

		whether they worked independently, and if applicable, details of automation tools used in the process.	
Effect measures	12	Specify for each outcome the effect measure(s) (e.g. risk ratio, mean difference) used in the synthesis or presentation of results.	5, Table 4 and Supplementary Information
Synthesis methods	13a	Describe the processes used to decide which studies were eligible for each synthesis (e.g. tabulating the study intervention characteristics and comparing against the planned groups for each synthesis (item #5)).	4-5, Tables 1-2
	13b	Describe any methods required to prepare the data for presentation or synthesis, such as handling of missing summary statistics, or data conversions.	4-5
	13c	Describe any methods used to tabulate or visually display results of individual studies and syntheses.	4-5
	13d	Describe any methods used to synthesize results and provide a rationale for the choice(s). If meta-analysis was performed, describe the model(s), method(s) to identify the presence and extent of statistical heterogeneity, and software package(s) used.	5
	13e	Describe any methods used to explore possible causes of heterogeneity among	5

		study results (e.g. subgroup analysis, meta-regression).	
	13f	Describe any sensitivity analyses conducted to assess robustness of the synthesized results.	NA
Reporting bias assessment	14	Describe any methods used to assess risk of bias due to missing results in a synthesis (arising from reporting biases).	Supplementary Information, SI Table S1
Certainty assessment	15	Describe any methods used to assess certainty (or confidence) in the body of evidence for an outcome.	Table 4
**RESULTS**			
Study selection	16a	Describe the results of the search and selection process, from the number of records identified in the search to the number of studies included in the review, ideally using a flow diagram.	6-7, PRISMA Diagram (Figure 1)
	16b	Cite studies that might appear to meet the inclusion criteria, but which were excluded, and explain why they were excluded.	-
Study characteristics	17	Cite each included study and present its characteristics.	8, 31
Risk of bias in studies	18	Present assessments of risk of bias for each included study.	Supplementary Information
Results of individual studies	19	For all outcomes, present, for each study: (a) summary statistics for each group (where appropriate) and (b) an effect estimate and its precision (e.g.	8-14, Supplementary information

		confidence/credible interval), ideally using structured tables or plots.	
Results of syntheses	20a	For each synthesis, briefly summarise the characteristics and risk of bias among contributing studies.	8-14, supplementary information
	20b	Present results of all statistical syntheses conducted. If meta-analysis was done, present for each the summary estimate and its precision (e.g. confidence/credible interval) and measures of statistical heterogeneity. If comparing groups, describe the direction of the effect.	8-14, Table 4
	20c	Present results of all investigations of possible causes of heterogeneity among study results.	NA
	20d	Present results of all sensitivity analyses conducted to assess the robustness of the synthesized results.	NA
Reporting biases	21	Present assessments of risk of bias due to missing results (arising from reporting biases) for each synthesis assessed.	-
Certainty of evidence	22	Present assessments of certainty (or confidence) in the body of evidence for each outcome assessed.	8-14
**DISCUSSION**			
Discussion	23a	Provide a general interpretation of the results in the context of other evidence.	16-18

	23b	Discuss any limitations of the evidence included in the review.	30-31
	23c	Discuss any limitations of the review processes used.	30-31
	23d	Discuss implications of the results for practice, policy, and future research.	16-18, 30-32
**OTHER INFORMATION**			
Registration and protocol	24a	Provide registration information for the review, including register name and registration number, or state that the review was not registered.	NA
	24b	Indicate where the review protocol can be accessed, or state that a protocol was not prepared.	NA
	24c	Describe and explain any amendments to information provided at registration or in the protocol.	NA
Support	25	Describe sources of financial or non-financial support for the review, and the role of the funders or sponsors in the review.	32-33
Competing interests	26	Declare any competing interests of review authors.	32-33
Availability of data, code and other materials	27	Report which of the following are publicly available and where they can be found: template data collection forms; data extracted from included studies; data used for all analyses; analytic code; any other materials used in the review.	32-33, Suppementary Information

From: [1] Page MJ, McKenzie JE, Bossuyt PM, Boutron I, Hoffmann TC, Mulrow CD, et al. The PRISMA 2020 statement: an updated guideline for reporting systematic reviews. BMJ 2021;372:n71. doi: 10.1136/bmj.n71

3. Supplemental Tables

a. Table S1. Modified Jadad Analysis for Methodological Quality.

StudyID	Authors	Criteria 1	Criteria 2	Criteria 3	Criteria 4	Criteria 5	Criteria 6	Criteria 7	Criteria 8	TOTAL
1	Affronti et al., 2018	0	0	0	0	1	1	1	1	**4**
2	Alexander et al., 2012	0	0	0	0	1	1	1	1	**4**
3	Alvarez et al., 2014	0	0	0	0	1	1	1	1	**4**
4	Assenat et al., 2021a	0	0	0	0	1	1	1	1	**4**
5	Assenat et al., 2021b	0	0	0	0	1	1	1	1	**4**
6	Bai et al., 2015	0	0	0	0	1	1	1	1	**4**
7	Balar et al., 2013	0	0	0	0	1	1	1	1	**4**
8	Basso et al., 2013	0	0	0	0	1	1	1	1	**4**
9	Bear et al., 2015	1	1	1	1	1	1	1	1	**8**
10	Buxo et al., 2018	0	0	0	0	1	1	1	1	**4**
11	Campbell et al., 2012	0	0	0	0	1	1	1	1	**4**
12	Chekerov et al., 2018	1	1	1	1	1	1	1	1	**8**
13	Chen et al., 2015	0	0	0	0	1	1	1	1	**4**
14	Chibaudel et al., 2019	0	0	0	0	1	1	1	1	**4**
15	Ciombor et al., 2014	0	0	0	0	1	1	1	1	**4**
16	Cremolini et al., 2020	1	1	0	0	1	1	1	1	**6**

17	de Vos et al., 2014	0	0	0	0	1	1	1	1	**4**
18	DeCensi et al., 2019	1	1	1	1	1	1	1	1	**8**
19	Deschenes-Simard et al., 2021	0	0	0	0	0	0	1	1	**2**
20	Donskov et al., 2018	1	1	0	0	1	1	1	1	**6**
21	Dowell et al., 2012	0	0	0	0	1	1	1	1	**4**
22	Dummer et al., 2012	0	0	0	0	1	1	1	1	**4**
23	Duvic et al., 2015	0	0	0	0	1	1	1	1	**4**
24	Fehr et al., 2020	1	1	0	0	1	1	1	1	**6**
25	Feliu et al., 2014	0	0	0	0	1	1	1	1	**4**
26	Fleming et al., 2014	1	1	0	0	1	1	1	1	**6**
27	Folprecht et al., 2016	0	0	0	0	1	1	1	1	**4**
28	Frizziero et al., 2019	0	0	0	0	1	1	1	1	**4**
29	Fuchs et al., 2019	1	1	1	1	1	1	1	1	**8**
30	Ghiaseddin et al., 2018	0	0	0	0	1	1	1	1	**4**
31	Ghiringelli et al., 2012	0	0	0	0	1	1	1	1	**4**
32	Goss et al., 2016	0	0	0	0	1	1	1	1	**4**
33	Gronberg et al., 2012	1	1	1	1	1	1	1	1	**8**
34	Guigay et al., 2015	0	0	0	0	1	1	1	1	**4**
35	He et al., 2020	0	0	0	0	1	1	1	1	**4**
36	Hirsch et al., 2017	1	1	0	0	1	1	1	1	**6**
37	Honecker et al., 2013	0	0	0	0	1	1	1	1	**4**

38	Hu et al., 2015	0	0	0	0	1	1	1	1	**4**
39	Idelevich et al., 2012	0	0	0	0	1	1	1	1	**4**
40	Ikemura et al., 2015	0	0	0	0	1	1	1	1	**4**
41	Ishida et al., 2015	0	0	0	0	1	1	1	1	**4**
42	Kakkos et al., 2020	0	0	0	0	1	1	1	1	**4**
43	Karavasilis et al., 2014	1	1	0	0	1	1	1	1	**6**
44	Kim et al., 2018	1	1	0	0	1	1	1	1	**6**
45	Kitayama et al., 2017	0	0	0	0	1	1	1	1	**4**
46	Konecny et al., 2015	0	0	0	0	1	1	1	1	**4**
47	Kottschade et al., 2013	1	1	0	0	1	1	1	1	**6**
48	Lang et al., 2012	1	1	0	0	1	1	1	1	**6**
49	Lara et al., 2016	1	1	0	0	1	1	1	1	**6**
50	Larsen et al., 2015	0	0	0	0	1	1	1	1	**4**
51	Lee et al., 2020	0	0	0	0	1	1	1	1	**4**
52	Maio et al., 2017	1	1	1	1	1	1	1	1	**8**
53	Makielski et al., 2015	0	0	0	0	1	1	1	1	**4**
54	Matsumoto et al., 2015	0	0	0	0	1	1	1	1	**4**
55	Michelsen and Sorensen, 2015	0	0	0	0	1	1	1	1	**4**
56	Mountzios et al., 2012	0	0	0	0	1	1	1	1	**4**
57	Nagane et al., 2012	0	0	0	0	1	1	1	1	**4**
58	Okines et al., 2013	1	1	0	0	1	1	1	1	**6**

59	Ottosson et al., 2020	0	0	0	0	1	1	1	1	**4**
60	Peeters et al., 2013	1	1	1	1	1	1	1	1	**8**
61	Pitz et al., 2015	0	0	0	0	1	1	1	1	**4**
62	Powell et al., 2013	0	0	0	0	1	1	1	1	**4**
63	Ramos et al., 2017	0	0	0	0	1	1	1	1	**4**
64	Reck et al., 2014	1	1	1	1	1	1	1	1	**8**
65	Reyes-Botero et al., 2018	0	0	0	0	1	1	1	1	**4**
66	Rivera et al., 2015	0	0	0	0	1	1	1	1	**4**
67	Saad et al., 2021	1	1	1	1	1	1	1	1	**8**
68	Salinaro et al., 2020	0	0	0	0	1	1	1	1	**4**
69	Salles et al., 2020	0	0	0	0	1	1	1	1	**4**
70	Seidel et al., 2012	0	0	0	0	0	1	1	1	**3**
71	Slagter et al., 2020	0	0	0	0	1	1	1	1	**4**
72	Sonpavde et al., 2011	1	1	1	1	1	1	1	1	**8**
73	Stevenson et al., 2012	0	0	0	0	1	1	1	1	**4**
74	Tahover et al., 2015	0	0	0	0	1	1	1	1	**4**
75	Tan et al., 2021	1	1	0	0	1	1	1	1	**6**
76	Tryfonidis et al., 2013	0	0	0	0	1	1	1	1	**4**
77	Tunio et al., 2012	0	0	0	0	1	1	1	1	**4**
78	Uetake et al., 2015	0	0	0	0	1	1	1	1	**4**
79	Usmani et al., 2019	1	1	0	0	1	1	1	1	**6**

80	Vaishampayan et al., 2014	0	0	0	0	1	1	1	1	**4**
81	Valle et al., 2021	1	1	1	1	1	1	1	1	**8**
82	Wolff et al., 2012	1	1	1	1	1	1	1	1	**8**
83	Yamazaki et al., 2016	1	1	0	0	1	1	1	1	**6**
84	Yardley et al., 2012	0	0	0	0	1	1	1	1	**4**
85	Zalcman et al., 2016	1	1	0	0	1	1	1	1	**6**
86	Baggstrom et al., 2018	1	1	1	1	1	1	1	1	8
87	Chavan et al., 2017	0	0	0	0	1	1	1	1	4
88	Duivenvoorden et al., 2016	0	0	0	0	1	1	1	1	4
89	Gay et al., 2010	0	0	0	0	1	1	1	1	4
90	Hong et al., 2012	0	0	0	0	1	1	1	1	4
91	Kang et al., 2012	0	0	0	0	1	1	1	1	4
92	Li et al., 2017	0	0	0	0	1	1	1	1	4
93	Martella et al., 2022	0	0	0	0	1	1	1	1	4
94	Matikas et al., 2016	0	0	0	0	1	1	1	1	4
95	Monk et al., 2018	0	0	0	0	1	1	1	1	4
96	Slavicek et al., 2014	0	0	0	0	1	1	1	1	4
97	Tachihara et al., 2020	0	0	0	0	1	1	1	1	4
98	Tewari et al., 2018	1	1	0	0	1	1	1	1	6
99	Yildiz et al., 2012	0	0	0	0	1	1	1	1	4

100	Lee et al., 2013	0	0	0	0	1	1	1	1	4
101	Reynes et al., 2016	0	0	0	0	1	1	1	1	4
102	Pinto et al., 2021	1	1	1	1	1	1	1	1	6

Criteria 1: Was the study randomised? (0 = no, 1 = yes)
Criteria 2: Was the method of randomisation appropriate (0 = no, 1 = yes)
Criteria 3: Was the study described as being blinded? (0 = no, 1 = yes)
Criteria 4: Was the method of blinding appropriate? (0 = no, 1 = yes)
Criteria 5: Was there a description of withdrawals and dropouts? (0 = no, 1 = yes)
Criteria 6: Was there a clear description of the inclusion/exclusion criteria? (0 = no, 1 = yes)
Criteria 7: Was the method used to assess adverse events described? (0 = no, 1 = yes)
Criteria 8: Was the method of statistical analysis described? (0 = no, 1 = yes)

b. Table S2. Publication Bias Scores for Studies.

StudyID	Authors	Publication Bias	Funding
1	Affronti et al., 2018	2	Amgen supported investigator-initiated trial, sponsored by the principal investigators of Affronti and Peters
2	Alexander et al., 2012	1	Grant from National Cancer Institute, one author consulted and had stock options in several biopharma companies
3	Alvarez et al., 2014	0	National Cancer Institute grants
4	Assenat et al., 2021a	2	Supported by Celgene
5	Assenat et al., 2021b	2	Work supported by CELGENE SAS

6	Bai et al., 2015	0	Funded by the Dutch Cancer Foundation, Swedish Cancer Society, Spanish Ministry of Economy and Competitiveness and Spanish Clinical Research Network
7	Balar et al., 2013	N/A	Undeclared
8	Basso et al., 2013	2	Austrian Fulvestrant Registry is supported by a grant from AstraZeneca Pharmaceuticals
9	Bear et al., 2015	2	Grant from Pfizer
10	Buxo et al., 2018	0	Supported by grant from NIH (America)
11	Campbell et al., 2012	0	No conflict of interest
12	Chekerov et al., 2018	0	Funded by research grants from the National Natural Science Foundation of China
13	Chen et al., 2015	0	No conflict of interest
14	Chibaudel et al., 2019	2	Clinical trial supported by Merck
15	Ciombor et al., 2014	1	Personal fees for various authors from Roche Pharma, Amgen, Sanofi, Bayer, Celgene, Lilly, etc
16	Cremolini et al., 2020	0	No conflict of interest
17	de Vos et al., 2014	1	Grant from National Cancer Institute, partly from OSI Pharmaceuticals and sanofi-aventis
18	DeCensi et al., 2019	2	Study supported by Seattle Genetics, all authors received research funding and some have equity ownership in the company
19	Deschenes-Simard et al., 2021	0	No conflict of interest
20	Donskov et al., 2018	2	Funded by Celgene
21	Dowell et al., 2012	2	Funded by Roche and Novartis, BEV provided by Roche
22	Dummer et al., 2012	2	Funded by Genentech, with research grants and compensation to multiple authors from Genentech and Lilly
23	Duvic et al., 2015	2	Eli Lilly Pharmaceutivals
24	Fehr et al., 2020	2	Funding from Pfizer, Sanofi-Aventis, NIH
25	Feliu et al., 2014	2	Supported by Hoffmann-La Roche

26	Fleming et al., 2014	2	Supported by Roche Farma, Spain
27	Folprecht et al., 2016	0	Grants from National Cancer Institute
28	Frizziero et al., 2019	N/A	Undeclared
29	Fuchs et al., 2019	0	Partially funded by American Society of Clinical Oncology
30	Ghiaseddin et al., 2018	2	Funded by Bristol-Myers Suibb and Howard I. Scher
31	Ghiringelli et al., 2012	1	Various authors have research funding and consulting relationship with pharma companies
32	Goss et al., 2016	0	Supported by National Institute of Health Grants and other Research Funds
33	Gronberg et al., 2012	0	Supported by NIH grants
34	Guigay et al., 2015	0	Funded by Norweigian Cancer Society, Nordic Cancer Union, etc
35	He et al., 2020	1	Supported by Copenhagen University Hospital and Danish Natinal Board of Health, an author has received research grants and speaking fees from Roche A/S and Merck
36	Hirsch et al.m 2017	2	Roche Pharma Schweiz
37	Honecker et al., 2013	N/A	Undeclared
38	Hu et al., 2015	2	Sanofi-aventis
39	Idelevich et al., 2012	2	Grants from Schering-Plough Corporation and Celgene Corporation
40	Ikemura et al., 2015	N/A	Undeclared
41	Ishida et al., 2015	N/A	Undeclared
42	Kakkos et al., 2020	2	Supported by Public Health Service grants and Eli Lilly Pharmaceutical Company
43	Karavasilis et al., 2014	0	No conflict of interest
44	Kim et al., 2018	2	Kyowa Kirin
45	Kitayama et al., 2017	2	Research dunging from Hedy Lee Kindler, Eli Lilly, Genentech, Roche
46	Konecny et al., 2015	2	Financial support from Celgene International

47	Kottschade et al., 2013	2	Funded by Novartis Pharmaceuticals
48	Lang et al., 2012	0	Funding from the National Cancer Institute Intramural Research Program
49	Lara et al., 2016	2	Work supported by F.Hoffmann-La Roche
50	Larsen et al., 2015	1	Supported by National Cancer Institute, in part by Biodesix and Genentech
51	Lee et al., 2020	0	Grants from the Natioanl Institute of Health
52	Maio et al., 2017	2	Supported by AstraZeneca
53	Makielski et al., 2015	2	Funded by AstraZeneca with multiple authors having funding from other pharmaceutical companies such as Eli Lilly, Novartis, Roche Laboratories
54	Matsumoto et al., 2015	0	Supported by National Cancer Research and Development Funds
55	Michelsen and Sorensen, 2015	2	Eli Lilly & Co
56	Mountzios et al., 2012	1	National Cancer Institute, but some authors are honoraria/stock ownership/research funding from Genentech
57	Nagane et al., 2012	2	Aventis and Roche Pharmaceuticals
58	Okines et al., 2013	0	Funded by Imperial Cancer Research Fund
59	Ottosson et al., 2020	0	Ministry of Health, Labour and Welfare of Japan grants
60	Peeters et al., 2013	2	Research funds from Eli Lilly and Co
61	Pitz et al., 2015	1	Capecitabine given by Roche
62	Powell et al., 2013	2	Partially funded by Glaxo SmithKline and Genentech Inc
63	Ramos et al., 2017	2	Partially funded by Glaxo SmithKline and Genentech Inc
64	Reck et al., 2014	2	Grant from Genentech
65	Reyes-Botero et al., 2018	2	Funding from Boehringer Ingelheim
66	Rivera et al., 2015	1	Research support from Celegene and honoraria

67	Saad et al., 2021	0	Grants from National Cancer Institute
68	Salinaro et al., 2020	2	Funded by Janssen Global Services, multiple authors receive benefits from pharma
69	Salles et al., 2020	0	No conflict of interest
70	Seidel et al., 2012	2	Supported by Taiho Pharma
71	Slagter et al., 2020	0	Supported by Department of Health and NIH grants and charity grants
72	Sonpavde et al., 2011	2	Aventis Pharmaceuticals
73	Stevenson et al., 2012	2	Supporrted by Hoffmann-La Roche and sanofi-aventis
74	Tahover et al., 2015	2	Supported by a grant from Genentech
75	Tan et al., 2021	0	No conflict of interest
76	Tryfonidis et al., 2013	2	Supported in part by Aventis Pharmaceuticals
77	Tunio et al., 2012	0	Supported by The Zena and Michael A. Wiener Research and Therapeutic Program in Bladder Cancer and National Cancer Institute Cancer Centre Core Grant
78	Uetake et al., 2015	N/A	Undeclared
79	Usmani et al., 2019	2	Merck Sharp & Dohme
80	Vaishampayan et al., 2014	2	Supported in part by Bristol-Myers-Squibb Inc
81	Valle et al., 2021	2	Grants from National Cancer Institute, and Genentech Inc and GPC Biotech
82	Wolff et al., 2012	0	Grant from National Cancer Institute
83	Yamazaki et al., 2016	2	Funded by Eli Lilly and Company
84	Yardley et al., 2012	1	Many honoraria, supported by West Japan Oncology Group
85	Zalcman et al., 2016	0	No conflict of interest
86	Baggstrom et al., 2018	0	Supported by National Cancer Institute
87	Chavan et al., 2017	0	No conflict of interest

88	Duivenvoorden et al., 2016	N/A	Undeclared
89	Gay et al., 2010	0	Supported by National Cancer Institute
90	Hong et al., 2012	1	Bevacizumab provided by Roche Korea
91	Kang et al., 2012	0	No conflict of interest
92	Li et al., 2017	N/A	Undeclared
93	Martella et al., 2022	0	No conflict of interest
94	Matikas et al., 2016	0	Funding from Cretan Association for Biomedical Research and Hellenic Oncology Research Group
95	Monk et al., 2018	1	Reception of clinical trial grants from numerous pharmaceutical companies
96	Slavicek et al., 2014	1	Unrestricted research grants from Roche, Merck and Amgen
97	Tachihara et al., 2020	1	Lecture fees and grants from Eli Lilly and Company
98	Tewari et al., 2018	2	Grants from Genentech
99	Yildiz et al., 2012	0	No conflict of interest
100	Lee et al., 2013	0	No conflict of interest
101	Reynes et al., 2016	2	Grant from Pfizer, Merck Sharp & Dohme
102	Pinto et al., 2021	2	Eli Lilly Italy

0 = Low potential for bias
1 = Moderate potential for bias
2 = High potential for bias

4. Supplemental Figures

a. Figure S1. Forest plot showing the estimated pooled prevalence of VTE in bladder cancer patients.

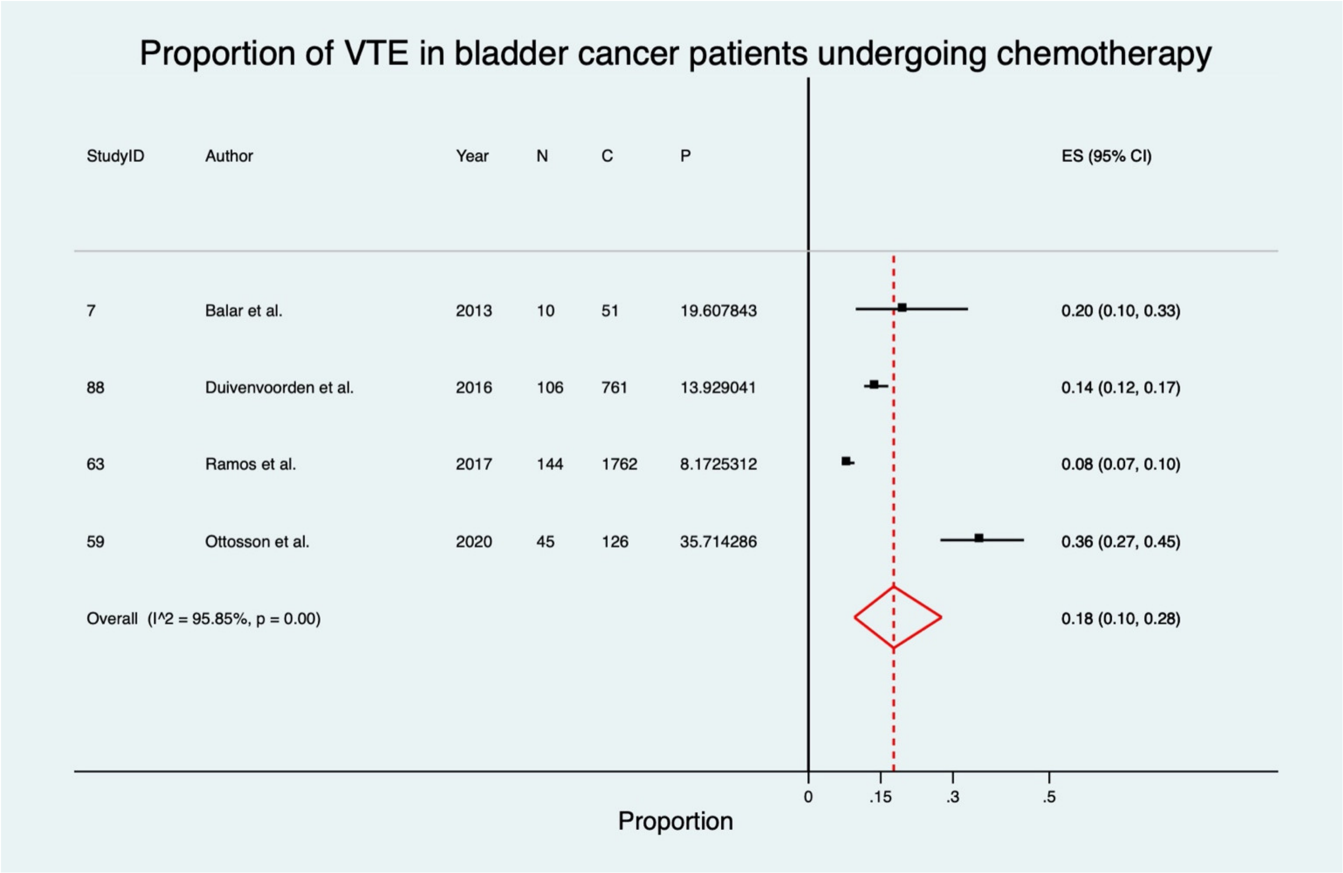

b. Figure S2. Forest plot showing the estimated pooled prevalence of VTE in brain cancer patients.

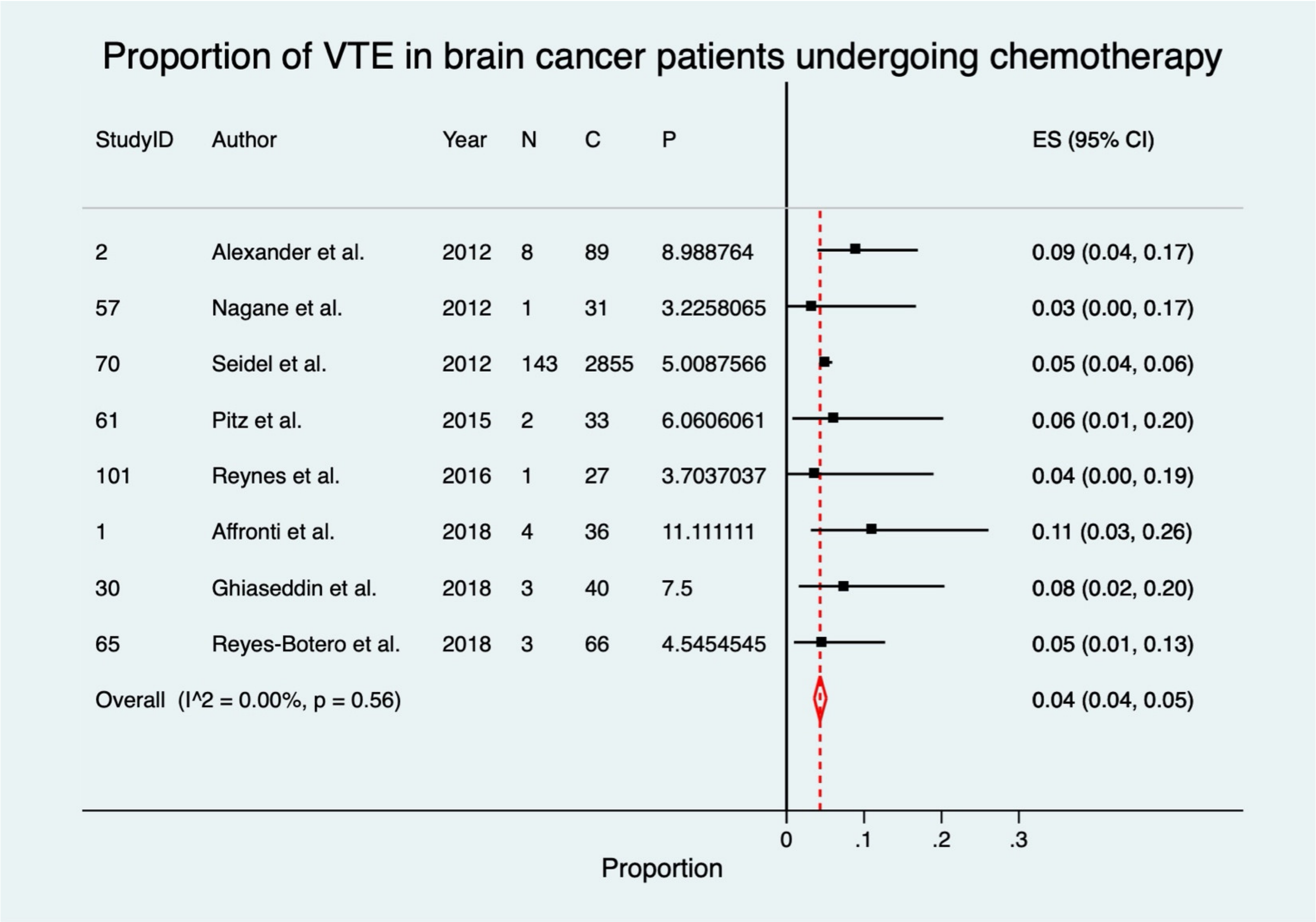

c. Figure S3. Forest plot showing the estimated pooled prevalence of VTE in breast cancer patients.

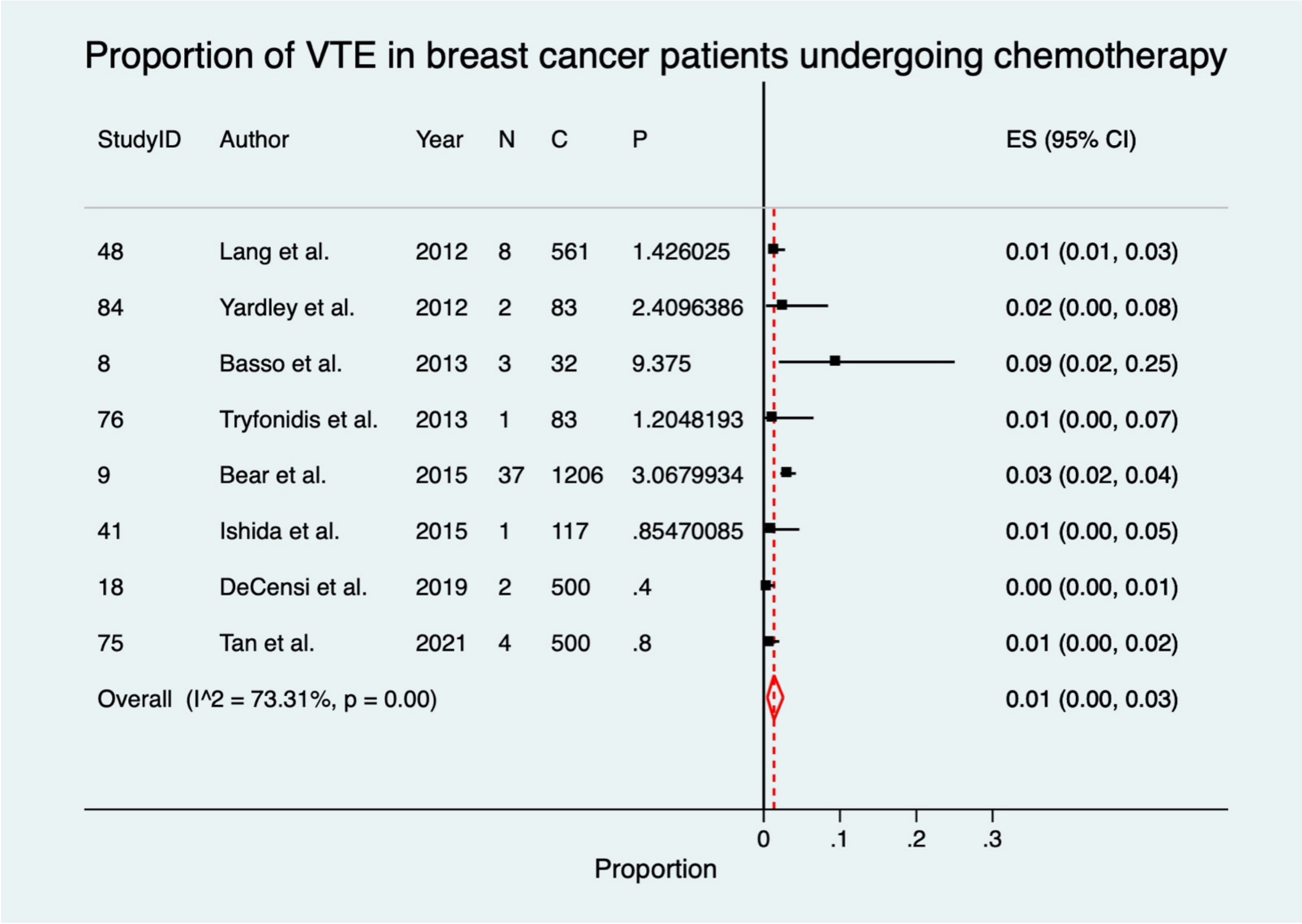

d. Figure S4. Forest plot showing the estimated pooled prevalence of VTE in colorectal cancer patients.

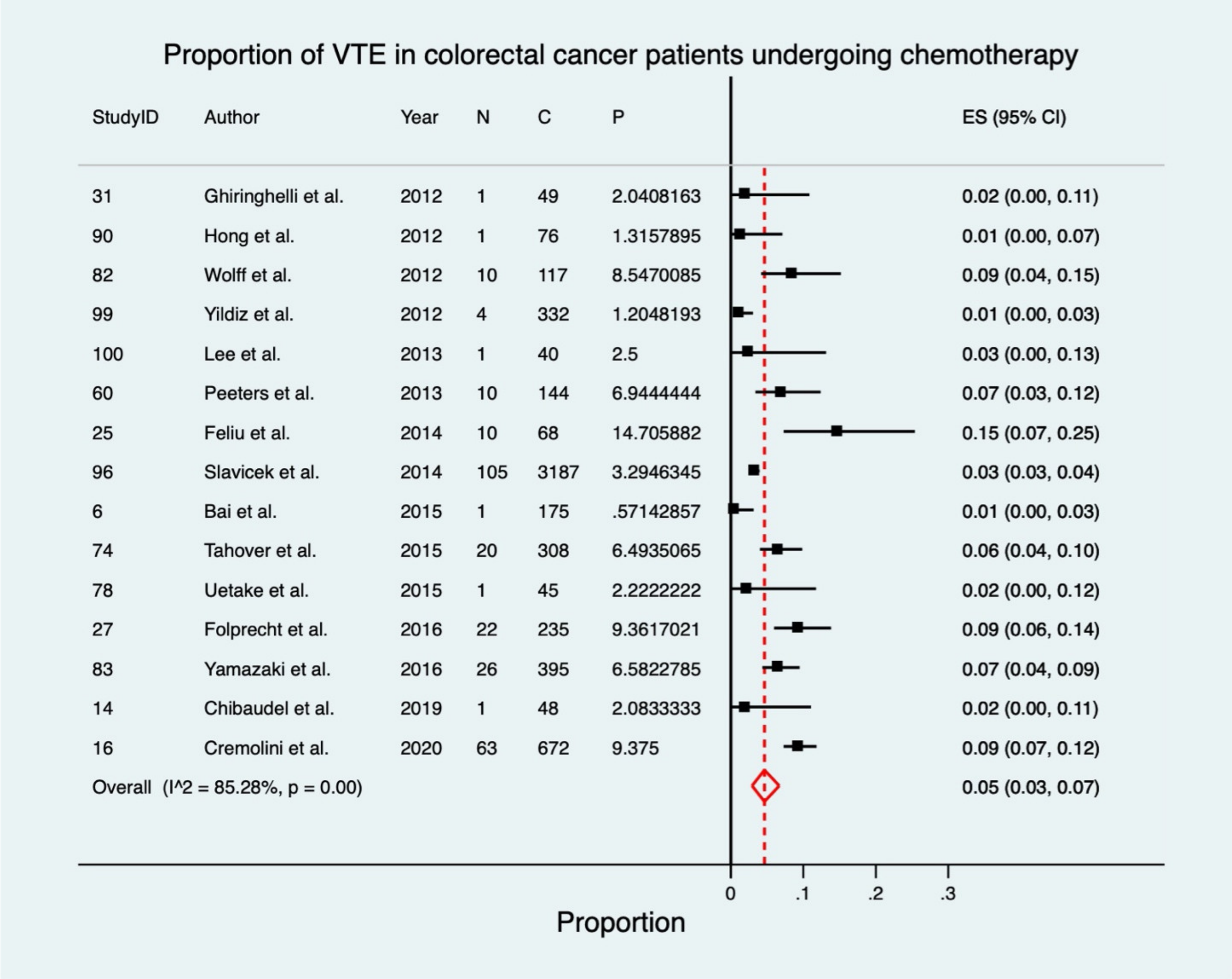

e. Figure S5. Forest plot showing the estimated pooled prevalence of VTE in gastric cancer patients.

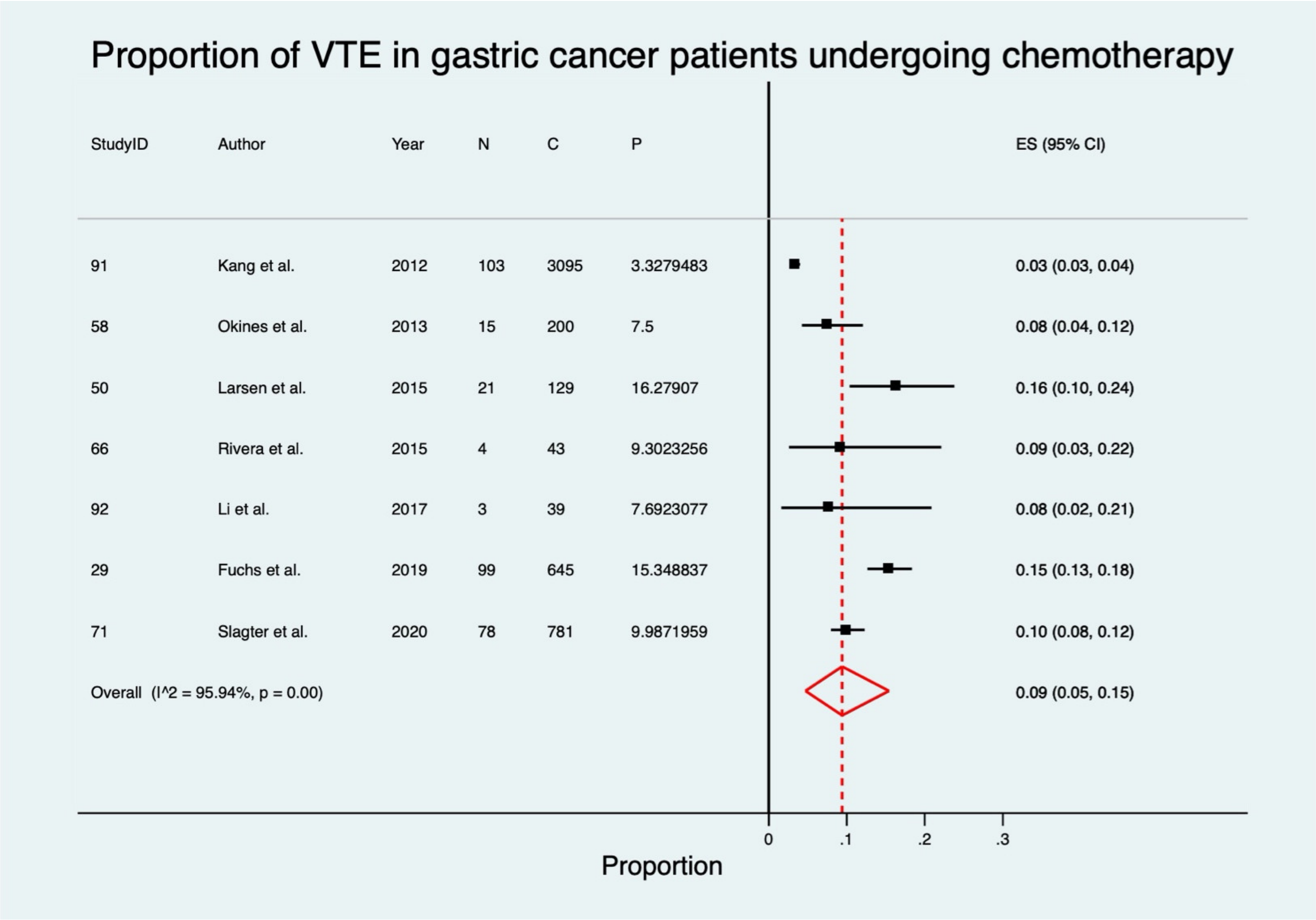

f. Figure S6. Forest plot showing the estimated pooled prevalence of VTE in lung cancer patients.

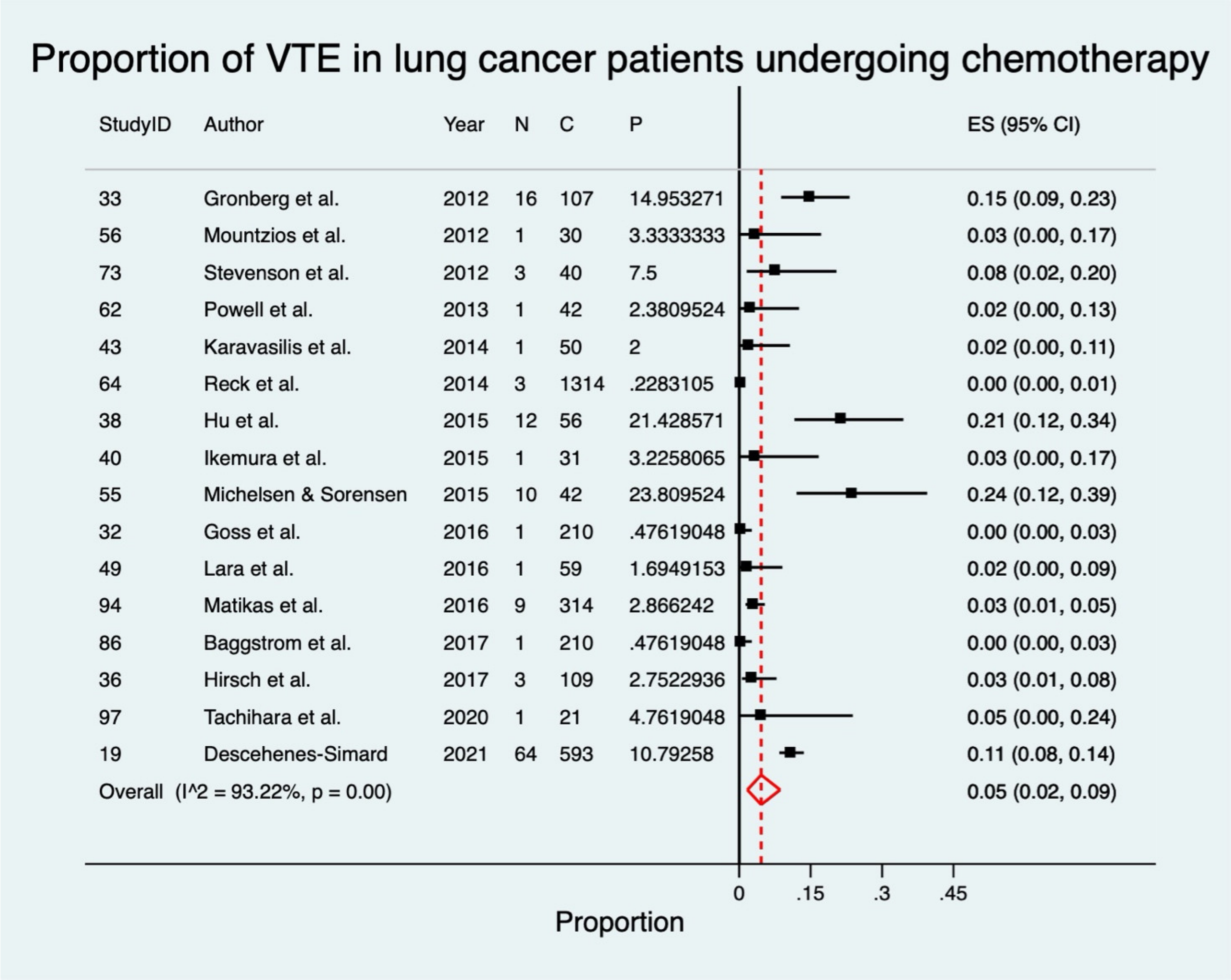

g. Figure S7.

Forest plot showing the estimated pooled prevalence of VTE in lymph cancer patients.

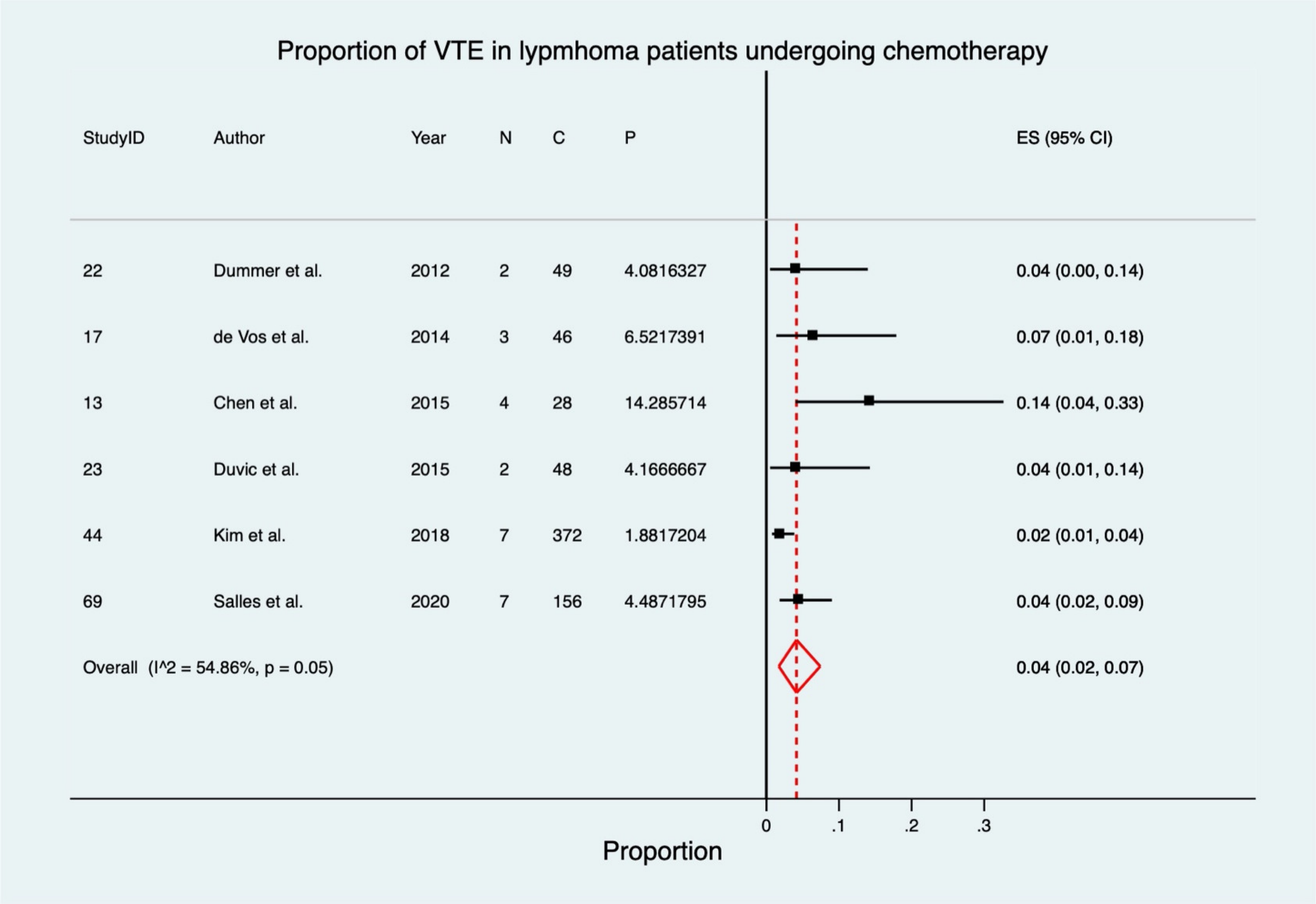

h. Figure S8. Forest plot showing the estimated pooled prevalence of VTE in mesothelial cancer patients.

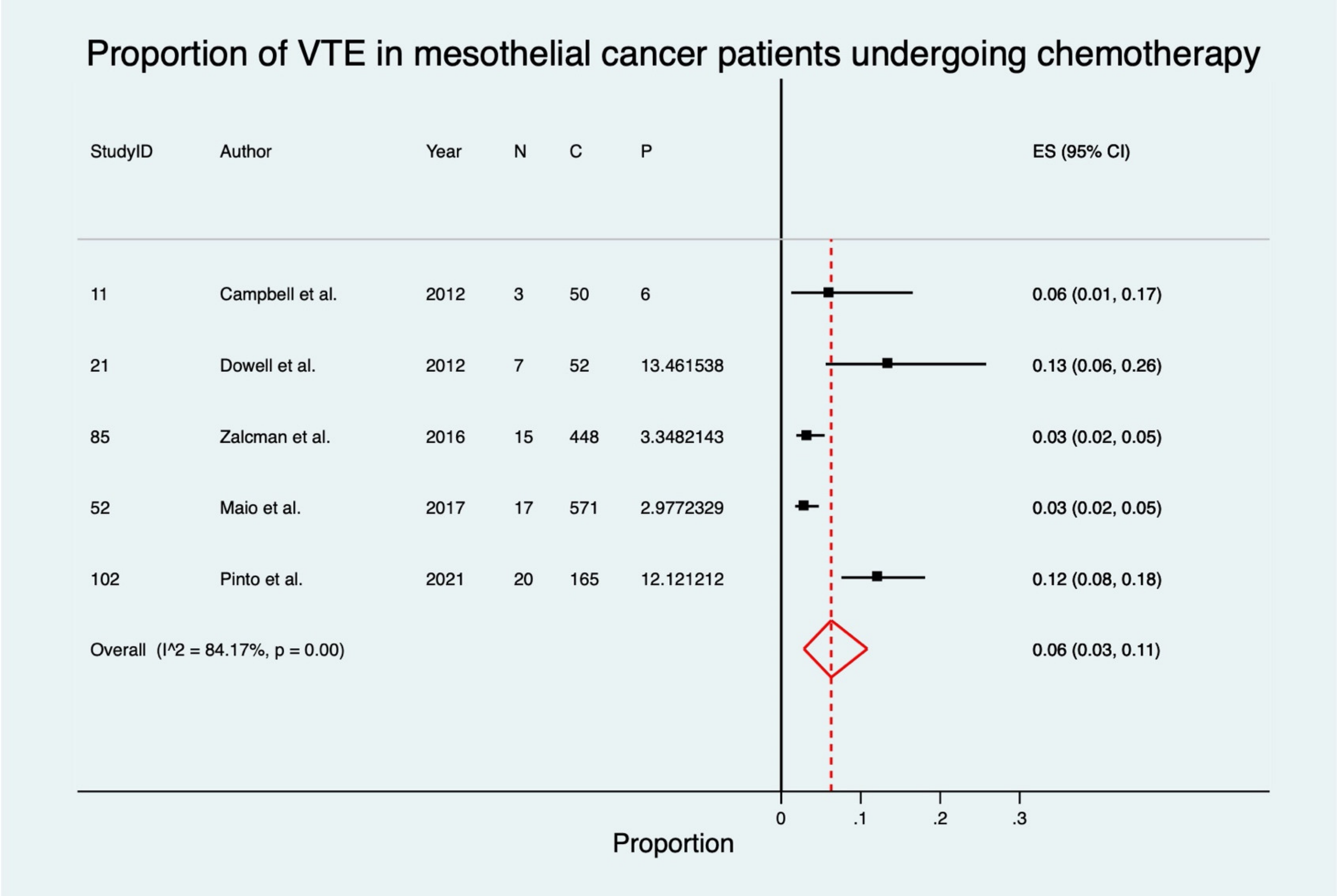
Proportion of VTE in mesothelial cancer patients undergoing chemotherapy
StudyID
Author
Year
N
C
P
ES (95% CI)
11
Campbell et al.
2012
3
50
6
0.06 (0.01, 0.17)
21
Dowell et al.
2012
7
52
13.461538
0.13 (0.06, 0.26)
85
Zalcman et al.
2016
15
448
3.3482143
0.03 (0.02, 0.05)
52
Maio et al.
2017
17
571
2.9772329
0.03 (0.02, 0.05)
102
Pinto et al.
2021
20
165
12.121212
0.12 (0.08, 0.18)
Overall (I^2 = 84.17%, p = 0.00)
0.06 (0.03, 0.11)
0
.1
.2
.3
Proportion

i. Figure S9. Forest plot showing the estimated pooled prevalence of VTE in ovarian cancer patients.

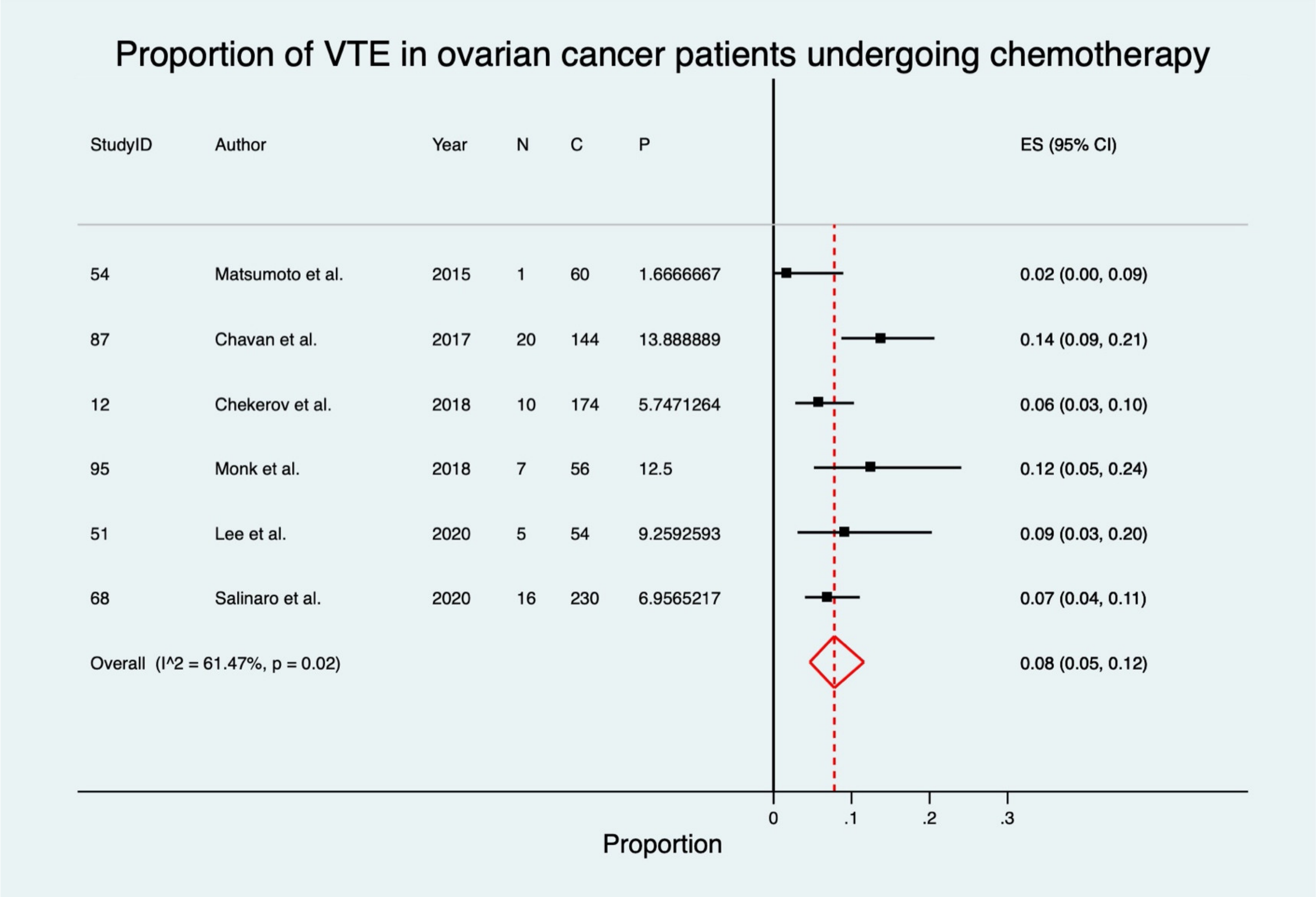

Supplement: Supplementary file 1 [file diagnostics-12-02954-s001.zip › diagnostics-1960491-supplementary (1).pdf]
